# Half-Yearly Report of Cases in Midwifery, Which Have Occurred in the Northern District of the London and Southwark Midwifery Institution

**Published:** 1833-02

**Authors:** C. Waller

**Affiliations:** Consulting Accoucheur to the above Institution, and Lecturer on Midwifery at 93, Bartholomew close, near St. Bartholomew's Hospital.


### MIDWIFERY.

*Half-yearly Report of Cases in Midwifery, which have occurred in the Northern District of the London and Southwark Midwifery Institution.* By C. Waller, m.d., Consulting Accoucheur to the above Institution, and Lecturer on Midwifery at 93, Bartholomew close, near St. Bartholomew's Hospital.

| 1832. | Number of Women delivered. | Sex of Children. | | Born Alive. | Stillborn. | Presentation. |
|---|---|---|---|---|---|---|
| | | Males. | Females. | | | |
| July ...... | 46* | 20 | 27 | 41 | 6 | 38 Natural<br>4 Breech<br>1 Arm & Funis<br>4 Face to Pubis |
| August.... | 37 | 16 | 21 | 34 | 3 | 36 Natural<br>1 Breech |
| September | 37 | 20 | 17 | 34 | 3 | 36 Natural<br>1 Breech |
| October... | 45 | 27 | 18 | 43 | 2 | 42 Natural<br>2 Face<br>1 Placenta |
| November | 52 | 30 | 22 | 50 | 2 | 51 Natural<br>1 Breech |
| December | 44 | 24 | 20 | 39 | 5 | 41 Natural<br>1 Funis & head<br>2 Face to Pubis |
| Total ... | 261 | 137 | 125 | 241 | 21 | * One case of twins. |

Several of the above, reported as Stillborn, were Premature Births.

Our institution has been remarkably free from disease since last report: there has not only been no death, but hardly a case of danger.

In one case, in which the head descended with the occiput forwards, it was found necessary to use the forceps; but the others required no manual interference. The more I see of these malpositions of the head, the more satisfied I am that, as a general rule, no interference on the part of the accoucheur is required, and, therefore, that the instructions for the rectification of such presentations, to be met with in some authors, are worse than useless. It is true, instruments may sometimes be required, yet their necessity occurs but rarely, and the injury likely to arise from the occasional use of the forceps is much less than that which would follow the invariable manual interference previously alluded to.

There was in one female a very tight adhesion of the placenta: some difficulty was experienced in its separation, the uterus having contracted closely around it. This circumstance, however, prevented hemorrhage, and thereby lessened the danger to the patient.

A very interesting case of convulsions occurred in a female in labour with her first child. She was perfectly senseless for many hours before the birth of the child, which presented the breech, and was born dead. The plan of treatment was bleeding from the arm before delivery, and the application of leeches, the administration of brisk purgatives, together with the constant use of an evaporating lotion afterwards: the result was perfectly successful.

There was great hemorrhage in the case of placenta presentation, but it had nearly subsided before I arrived at the house. The breech of the child presented, and my pupil, Mr. Newth, whose patient it was, had succeeded in bringing down the feet, whilst the messenger was despatched for me; so that I had merely to look on whilst he completed the delivery. The case was conducted in a manner highly creditable to him. The patient, having no bedstead, was obliged to lie on the floor, which rendered the operation more difficult than it would otherwise have been.

In the arm presentation, the liquor amnii came away at the beginning of the labour; but the uterus remaining very inactive, no great difficulty was experienced, although turning was not effected until forty-eight hours had elapsed.

*Bartholomew Close; January* 1833.

